# EEGs Vary Less Between Lab and Home Locations Than They Do Between People

**DOI:** 10.3389/fncom.2021.565244

**Published:** 2021-02-16

**Authors:** Kaare B. Mikkelsen, Yousef R. Tabar, Christian B. Christensen, Preben Kidmose

**Affiliations:** Department of Electrical and Computer Engineering, Aarhus University, Aarhus, Denmark

**Keywords:** electroencephalogram, home recording, inter subject variability, intra subject variability, ear-EEG

## Abstract

Given the rapid development of light weight EEG devices which we have witnessed the past decade, it is reasonable to ask to which extent neuroscience could now be taken outside the lab. In this study, we have designed an EEG paradigm well suited for deployment “in the wild.” The paradigm is tested in repeated recordings on 20 subjects, on eight different occasions (4 in the laboratory, 4 in the subject's own home). By calculating the inter subject, intra subject and inter location variance, we find that the inter location variation for this paradigm is considerably less than the inter subject variation. We believe the paradigm is representative of a large group of other relevant paradigms. This means that given the positive results in this study, we find that if a research paradigm would benefit from being performed in less controlled environments, we expect limited problems in doing so.

## 1. Introduction

With the advent of smart devices and wearable technologies, real life EEG recordings are getting increasingly feasible and potentially useful (Debener et al., 2012, 2015; Mullen et al., 2015). Applications include diagnosing and monitoring of epileptic patients (Gilliam et al., 1999; Askamp and van Putten, 2014; Zibrandtsen et al., 2017), decoding of auditory attention (Mirkovic et al., 2015; O'Sullivan et al., 2015; Das et al., 2018), brain-computer interfaces (Birbaumer and Cohen, 2007; De Vos et al., 2014), sleep monitoring (Shambroom et al., 2012; Younes et al., 2017; Mikkelsen et al., 2019), and monitoring of human behavior in extreme situations, such as cave exploration or space travel (Mogilever et al., 2018), to name a few. With the ongoing SARS-CoV-2 pandemic, the simple need to continue clinical investigations and EEG research outside laboratories has been added to the list.

However, given that the majority of existing EEG literature deals with single measurements on many subjects, there is limited data on the likely changes to results, or any decrease in data quality, that would come about from performing multiple measurements on the same subjects, in different locations, possibly outside of the laboratory and the immediate control of the investigator.

Looking at the literature, we find some studies focusing on intra- and inter-subject variability in the lab. Corsi-Cabrera et al. (1997, 2007) looked at patterns of correlation in scalp EEG in women, and found stable differences between subjects. Stastny et al. (2014) showed that inter-subject variability in the μ-rhythm could be used to identify subjects between sessions. Dalebout and Robey (1997) showed in 1997 that the P300 response varies more between subjects then within them, and in the late 80'ies Lauter et al. showed extensively that audiological responses follow the same pattern (Lauter and Karzon, 1990a,b).

More recently, Poulsen et al. (2017) showed that the amount of intersubject variability in a classroom setting could be used to gauge group engagement. Given that this is in itself an example of EEG recordings taken out of the laboratory setting, the comparison is particularly interesting.

Finally, Shen and Lin (2019) studied both inter and intra-subject variation in EEG during emotional responses. They found substantial inter- and intra-subject variation, not unlike what we show here.

In this study we present a paradigm designed to be both doable outside a laboratory, as well as reasonably comparable to a broad class of EEG paradigms. Second, we quantify the relationship between inter-subject, intra-subject (inter-session) and inter-location variability for this paradigm, and for each individual response invoked by it.

We tasked 20 subjects with performing the instructions in a 3-min long video on 8 separate occasions—4 in our EEG laboratory and 4 in their respective homes. All while wearing a combination of EEG, EOG, and chin EMG electrodes. By comparing the variation contribution from the different sources, we find clear inter-subject variability in all measures, and only little location-dependence. However, we do find that the unexplained variance generally increases for recordings performed outside the laboratory.

## 2. Methods

### 2.1. Setup

The recording setup consisted of 25 iridium oxide electrodes (Kappel et al., 2019), connected to a TMSi Mobita amplifier.

The TMSi mobita amplifier is a mobile EEG amplifier with 24 bit resolution, individually shielded inputs, less than 0.4 micro V RMS noise in the 0.1–10 Hz band, greater than 10 GΩ input impedance, and greater than 100 dB CMRR.

**Figure 1:**
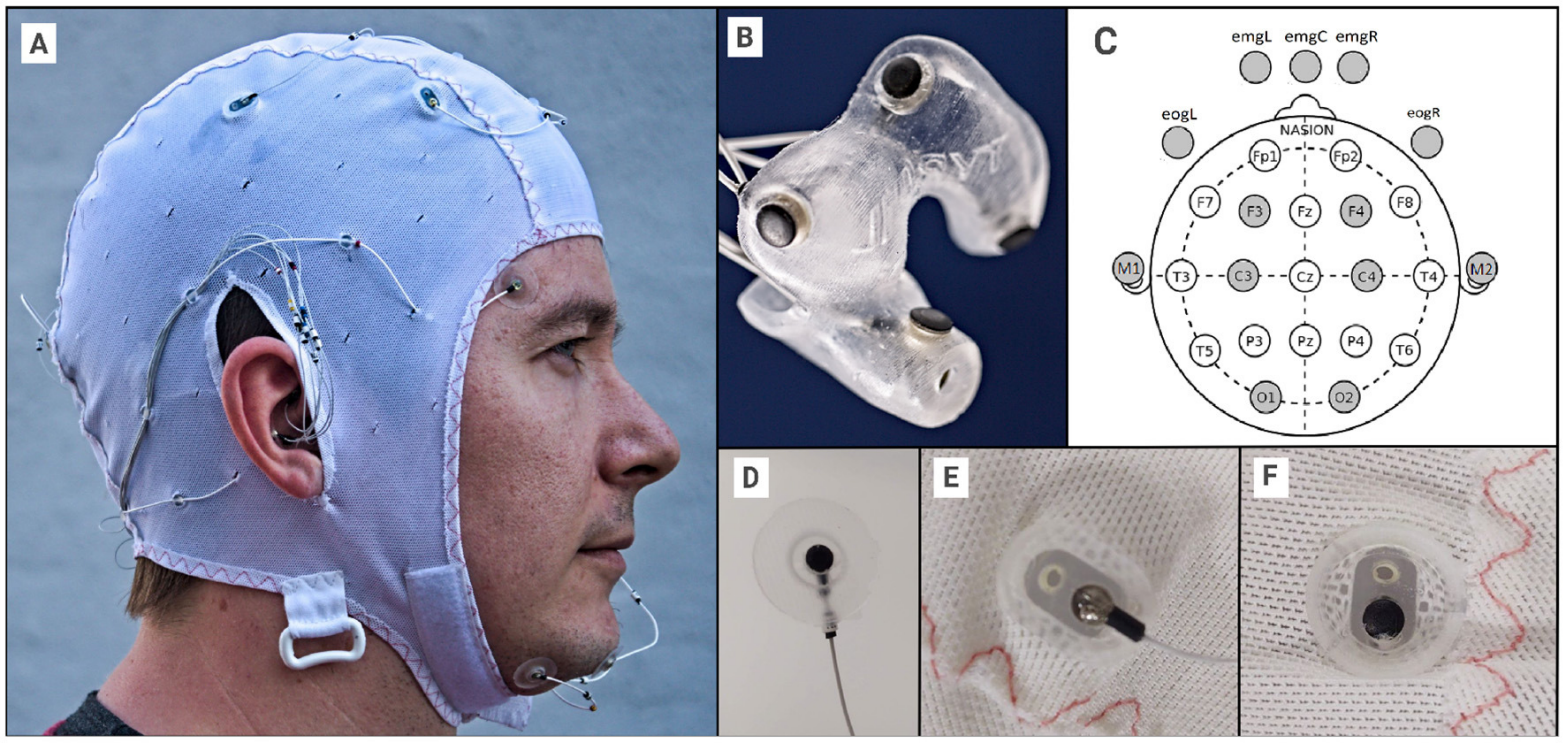
**(A)** The EEG setup, with EEG cap, face electrodes and ear-EEG plugged in. **(B)** Close-up for single ear-EEG earplug (left in this case). **(C)** 10-20 reference diagram, showing the used scalp electrodes, including the 5 facial electrodes. **(D)** The EOG, EMG and M1/M2 electrodes were held in custom silicone holders. **(E)** Cap electrode holder from the outside. **(F)** Cap electrode holder from the inside. The diameter of the electrode is 3.5 mm and the diameter of the “cup” is approximately 10 mm. Written informed consent was obtained from the individual for the publication of any potentially identifiable images or data included in this article.

The setup was a combination of ear-EEG (Mikkelsen et al., 2015), scalp EEG, EOG, and EMG electrodes: 12 positions within the ears (6 per ear, see Figure 1), 3 chin EMG electrodes, two EOG electrodes and 8 scalp electrodes (M1,M2,C3,C4,F3,F4,O1,O2) (see Figure 1). All electrodes were essentially identical, as seen in Figures 1B–F. To ensure good connections, all electrodes outside the ears (13 in total) were treated as wet electrodes and received electrode gel (Elefix from Nihon Kohden for electrodes on the scalp, Ten20 from Weaver and Company for face and mastoid). To ensure reliable connections on the scalp, liberal amounts of Elefix gel were used, in particular for subjects with long or curly hair (however, it was ensured that bridging between gel patches never occurred). All electrodes were embedded in soft silicone holders, and the cap was an EASYCAP EEG cap (EASYCAP GmbH, Germany), modified in-house. Ear-EEG ear pieces were costum made for the individual, ensuring a good and stable connection.

It is worth noting that the electrode gels were specifically chosen because they do not dry out (they are not hydrogels). Furthermore, as the dry electrodes, by design, can not dry out either, the electrode connections in the whole setup should be expected to be very stable over time.

The signals were sampled at 500 Hz, and a disposable electrode (Ambu, White Sensor, WS-00-S) placed on the neck was used as ground. The Mobita amplifier always uses an average reference during recording.

The EEG laboratory used in this study was a dedicated room in which we have successfully performed a host of different electrophysiological recordings, and which is used in teaching electrophysiological methods. During the recordings, no other activities took place in the room, leading to a quiet setting. The room has a sufficiently low amount of background electrical noise that additional electrical shielding has not been necessary.

The study was reviewed and approved by the Central Denmark Region Committees on Biomedical Research Ethics (Ref. nr. 1-10-72-413-17) as well as the Danish Medicines Agency (ref. nr. 2017111085).

### 2.2. Paradigm

Each recording consisted of two portions—one in a controlled laboratory in an EEG lab, and one in the subject's own home. The electrode setup was performed in the laboratory immediately prior to the first portion, and then kept on until the second portion at home. The setup was performed by an experienced EEG experimenter. The subjects were informed that they could take out the ear-EEG electrodes after the lab measurement and then put them back in before the home measurement. This option was used a total of 14 times (out of 80 possible). The time difference between first and second portion was, on average, 5 h and 9 min.

Each subject had 4 recording days, meaning that the video was viewed 8 times by each subject. On average, there was a 19 day gap between each recording day, though with considerable variation (25% were below 7 days, 51% below 14 days).

#### Behavioral Paradigm

While watching a video (accessible at https://www.youtube.com/watch?v=4Uh2UeDzizk), the participants were instructed to:

- Perform 5 jaw clenches
- Alternate between 20 s of open and closed eyes, with two repetitions
- Perform rhythmic, lateral eye motions.

The video takes 3 min and 9 s.

**Figure 2:**
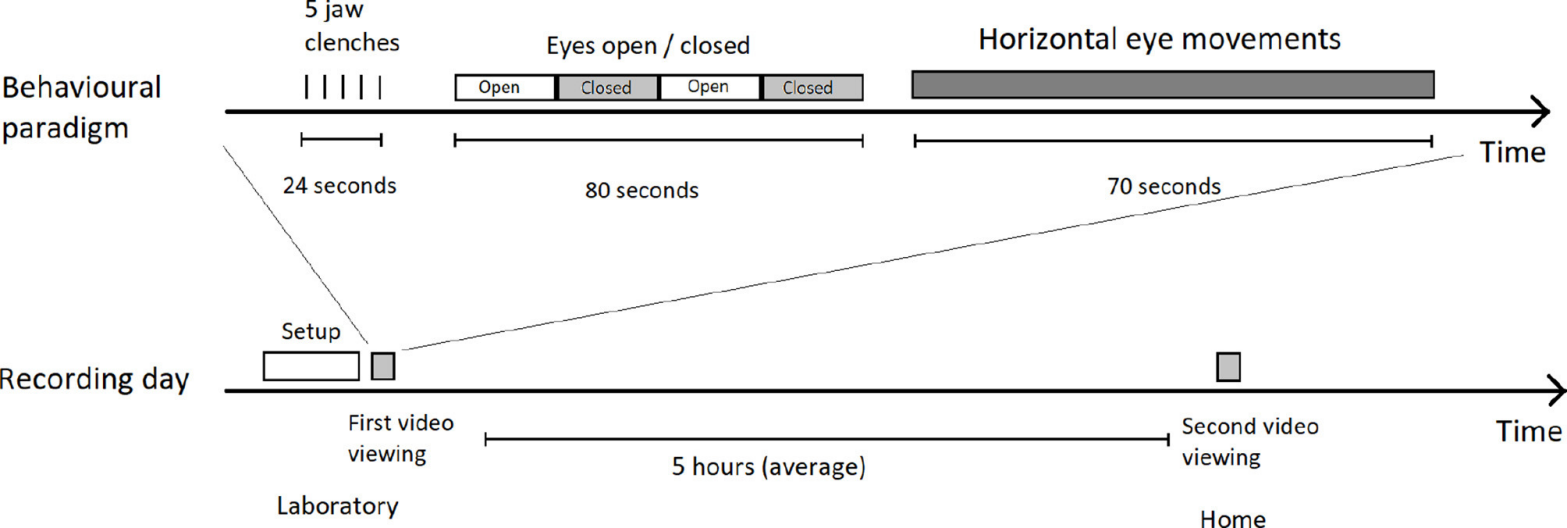
Overview of a recording. **Top** shows the structure of the video and included instructions, **bottom** shows how the video was seen twice on the same day by the participant, once immediately after recording setup, and the second time several hours later in their own home (still wearing the recording equipment). The same video, with the same instructions, is viewed every time.

Please see Figure 2 for a detailed diagram of experimental procedure.

It is worth noting that we took inspiration from the typical paradigms used for quality control of EEG setups in a clinical setting. This means that the expected responses in the recording are easy to recognize, and it is possible to positively identify whether the participant correctly followed the instructions.

### 2.3. Cohort

Twenty subjects were recruited, with ages between 22 and 36, mean 25.9 years. Thirteen were female. Subjects were a mixture of lay people (4), engineering students (15) and researchers (1). In total, 3 out of the 20 subjects could be considered to have prior EEG experience.

Subjects received monetary compensation for their participation.

### 2.4. Preprocessing

The timing between the EEG and the video instructions was determined by identifying the beginning of the lateral eye movements in the EOG, and extracting data up to 105 s prior to that as well as up to 85 s after.

**Figure 3:**
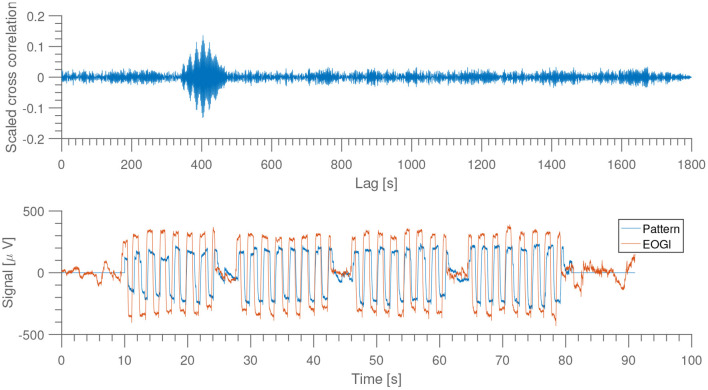
**Top**: scaled version of the cross correlation between recorded signal from EOGl electrode and a sample recording used as “pattern.” **Bottom**: comparison of EOGl and sample pattern at highest cross correlation. From subject 1, night 1, home recording.

As shown in Figure 3, the eye-movement dominated portion of the EEG is quite clear, and by using this it is possible to get an automatic, reproducible alignment at sub-second precision.

In preparing all EEG recordings, we employed a mixture of automatic and manual artifact rejection:

1. All channels in all recordings were band pass filtered to only keep activity in the 0.3 to 100 Hz band. This was done using a Hamming windowed sinc FIR filter of order 5500 [as implemented in EEGLAB (Delorme and Makeig, 2004)].
2. Instances where a single electrode had an amplitude larger than 350 μ*V* were identified as artifacts, and the samples from that particular electrode was set to “nan” for a 2-s window around the event.
3. Finally, we used the fact that the ear electrodes have a high degree of redundancy between them, meaning that it should be possible to predict most of the signal from a healthy electrode using the signal from the neighboring electrodes. This was implemented by rejecting any ear electrode that had less than 0.4 Pearson correlation between itself and it's projection into the space of all other ear electrodes. The value of 0.4 was chosen to match rejection through visual inspection.

Due to the quite large signal amplitudes evoked by the eye-movement portion, the amplitude-thresholding was skipped for that part of the recordings.

Finally, the recordings were checked manually, to spot any additional channels to reject.

## 3. Data Modeling

To maximize clarity and relevance of the analysis, for each part of the analysis of the paradigm, we focus on specific choices in modeling and specific electrodes (rather than report outputs from all possible electrode configurations). Thus, we do not restrict ourselves to a specific choice among the 25 electrodes, but have instead chosen to use the derivations that are most relevant for each sub analysis. See below for further details.

### 3.1. Jaw Clench Modeling

**Figure 4:**
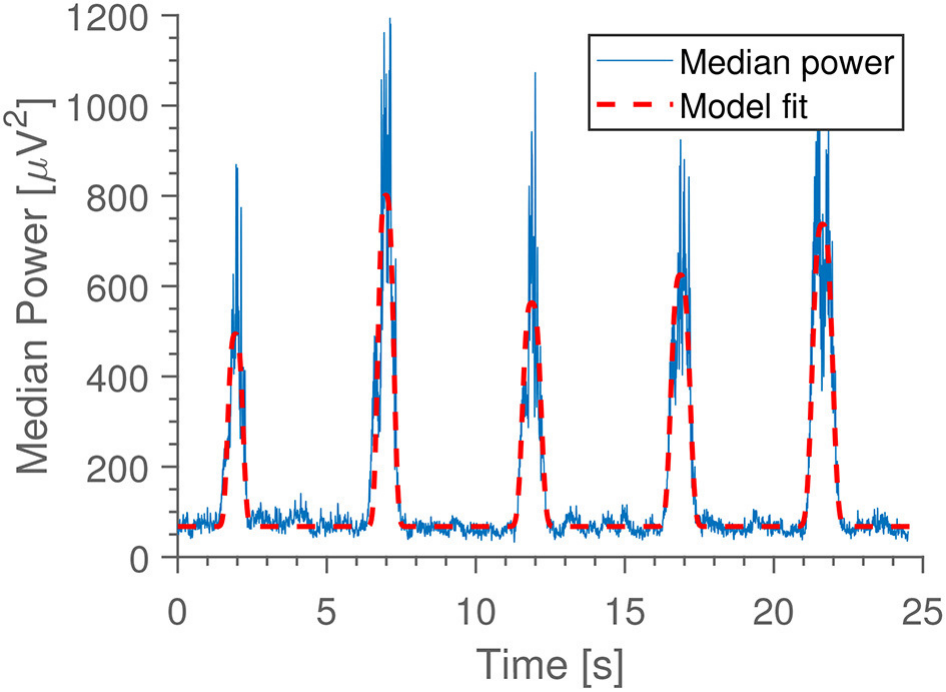
Example of the MEG model [*f* in (1)] fitted to median gamma power.

Jaw clenches are characterized by an increase in power at high frequencies (40–1,000 Hz), seen easily in electrodes placed close to the jaw muscles. Because of this, we extracted the power in the 40–80 Hz band for each ear electrode in windows of 0.5 s long, and calculated median power across electrodes for each window. By fitting the function:

(1) $$\begin{array}{l} f\left(t\right)=c+\overset{5}{\underset{i=1}{\sum }}a_{i}\cdot e^{{\left(t-c_{i}\right)}^{2}/w_{i}^{2}} \end{array}$$

we may estimate the intensity of the clenching by the extracted amplitudes, *a*_*i*_. Here, *c, a*_*i*_, *c*_*i*_, *w*_*i*_ are all free parameters determined through least squares fitting, and the index *i* represents the five jaw clenches (such that *c*_*i*_ is the timing and *w*_*i*_ the width of clench *i*). See Figure 4 for an example. Prior to power estimation, the ear electrodes were referenced to their own average.

### 3.2. Alpha Power

The occipital alpha oscillation is present all over the head, but it is seen clearest in the occipital electrodes. Therefore, we estimated the power in the 8–12 Hz band averaged over electrodes O1 and O2, during the eyes open/closed portion of the paradigm. The two electrodes were referenced to the average of the scalp electrodes.

### 3.3. EOG Content

One way to characterize the EEG recordings is to specifically look at the different noise sources, to see whether they influence the recordings in the same manner across locations. An example of this is extracting eye movements using a single-sided ear-EEG device, which, besides its use in characterizing EEG recordings, could also be considered as a means to estimate visual attention (Favre-Félix et al., 2019).

To test this, we used a linear model, similar to what was used in Mikkelsen et al. (2017), to predict the activity in the EOGr-EOGl derivation during the “horizontal eye movements” portion of the behavioral paradigm. More precisely, the 70 s of eye movements were partitioned into two 35 s intervals, and a linear model (with a constant term) was trained to mimic the EOG activity using only the left or right ear electrodes. The model trained on the first 35 s was then applied to the second 35 s, and vise versa. For each ear, performance was recorded as the Pearson correlation between actual EOG signal and predicted.

From this point, we shall exclusively refer to this correlation as the “prediction quality.”

### 3.4. Resting State Power Levels

It is generally interesting to know how the spontaneous, or “background” variation in the EEG data differs between locations and subjects, to infer whether paradigms known from recordings in the lab can be performed at home. In practice, this would be the main contribution to the “noise floor” in an ERP measurement.

We estimate the resting state EEG power by measuring the power for various electrode combinations (M1, avr. left ear, avr. right ear, C3, C4, F3, F4, all referenced to M2), and presenting both the full spectrum (up to 100 Hz) as well as the behavior of the average power in the 30–100 Hz band (excluding 50 Hz). We only used the “eyes open/closed” portion of the recordings for this analysis, and the power spectrum was calculated using Welch's algorithm with 3-s window widths.

### 3.5. Linear Mixed Models

A central question is to which extent variation in the independent variables causes variation in the results; it is very helpful when designing an experiment to know what alteration of study design may risk drowning out the signal.

In this study, the three most interesting sources of variation are that caused by adding subjects, that caused by repeating measurements, and that caused by changing location. In short, for *m* = 1, ..., 20 subjects, *n* = 1, ..4 repetitions and *l* = 1, 2 locations, the 80 observations, *y*_*mnl*_, may be described as:

(2) $$\begin{array}{l} y_{mnl}\sim c+L_{l}+S_{m} \end{array}$$

(3) $$\begin{array}{l} S_{m}\sim N\left(\mu_{m},\sigma_{1}^{2}\right) \end{array}$$

(4) $$\begin{array}{l} \mu_{m}\sim N\left(\mu ,\sigma_{2}^{2}\right) \end{array}$$

where *N*(μ, σ^2^) is a normal distribution with mean μ and variance σ^2^. In this terminology, $\sigma_{1}^{2}$ represents the intra subject variation, and $\sigma_{2}^{2}$ the inter-subject variation.

We apply our framework to the data using linear mixed effects models, letting “subject identity” be a “random effect” and everything else “fixed effects.” Since we are doing the calculations in MATLAB (using fitlme), we describe the five models using Wilkinson notation (Wilkinson and Rogers, 1973):

**Jaw Clench, alpha power**:

*y* ~ 1 + location + (1|subject).

**EOG content**:

*y* ~ 1 + location + side-of-head + (1|subject).

**Resting power levels**:

*y* ~ 1 + location + channel + (1|subject).

Note that we add either “channel” or “side-of-head” dependencies in the last two, so as not to unduly add to the “intra subject variation.”

By fitting the mixed effects models to the data, we can define the inter-subject, intra-subject and inter-location variation in the following ways:

**Inter-subject:** *std*(μ_*i*_), where μ_*i*_ is the average response from subject *i*.
**Intra-subject:** The residual error, or root mean square error of the model fit. This can also be thought of as “day-to-day variation.”
**Inter-location:** So as not to compare fixed-effects offsets to sums of squared errors, we represent the inter-location variation by the square root of the “squared error” term for the location-term, as calculated by “fitlme” in MATLAB.

We calculate these for each of the analyses described above, and rescale them such that the largest source of variation is 1 for each comparison, to make it easier to compare results from different paradigms.

### 3.6. Analysis of Location Influence

To specifically quantify the effects of doing measurements in multiple locations, we calculate *p*-values for both the differences in mean values between laboratory and home measurements, as well as the unexplained variances for each location (the “noise”).

*p*-values for the significance of mean differences are extracted from the mixed linear model fits (the “ANOVA” field in the fitlme output) and for variation differences, we use permutation testing (by pairwise permutation of the location information) to estimate the probability of getting a greater difference than the one observed. All *p*-values are for two-tailed tests.

## 4. Results

### 4.1. Data Quality

On five occasions, the behavioral data were lost. This happened on four occasions in the lab, and on one occasion at home. In the lab, it was due to human error in mismanagement of the recording, at home, the subject simply forgot to do it.

The automatic and manual artifact rejection resulted in 9% of the data samples being rejected (11% in lab, 7% at home). 1.5% of the data was rejected in the manual step. Viewing the setup as a whole, 2% of the time points were rejected (meaning that at 98% of the time, at least one electrode was recording). These numbers for lab vs. home were 4.6 vs. 0.2%. We have excluded the 5 missing recordings when calculating this.

### 4.2. Jaw Clenching

**Figure 5:**
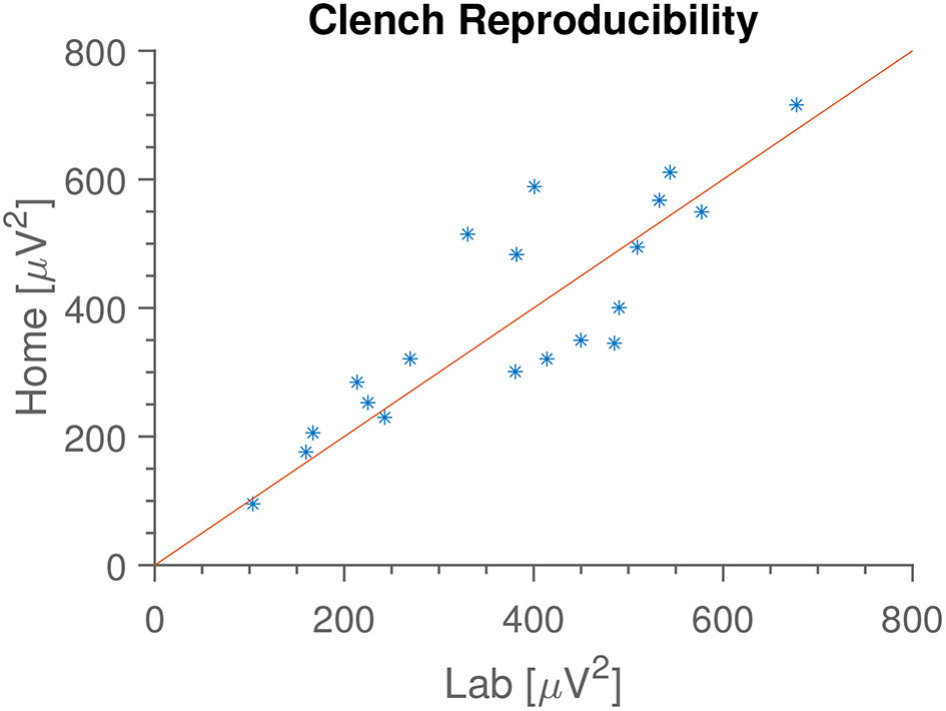
Median median clench amplitude for each subject, in the two locations. Meaning that we calculate the median clench for each recording, and then find the median of those values. We see that median clench is very consistent between home and lab environments, and that the value varies between subjects. Identity line included for reference.

In Figure 4 is seen an example of the extracted median gamma band power. The most common deviations from this pattern are either a missing first peak (some subjects forgot to do the first clench) or some disturbance occurring halfway through (coughing, other movements). In Figure 5 is shown the “median of medians” peak amplitude, meaning that first the median amplitude is calculated for each recording, and subsequently the median is taken for each subject's recording date. We see that this observable is very consistent within subjects between home and lab settings. Since the peak amplitude is influenced by how vigorously the subjects clench, we interpret this to mean that subjects were equally enthusiastic with and without direct supervision; they did not just go through the movements when at home, but strove to do the task as well as they had done in the laboratory.

### 4.3. Alpha Power

**Figure 6:**
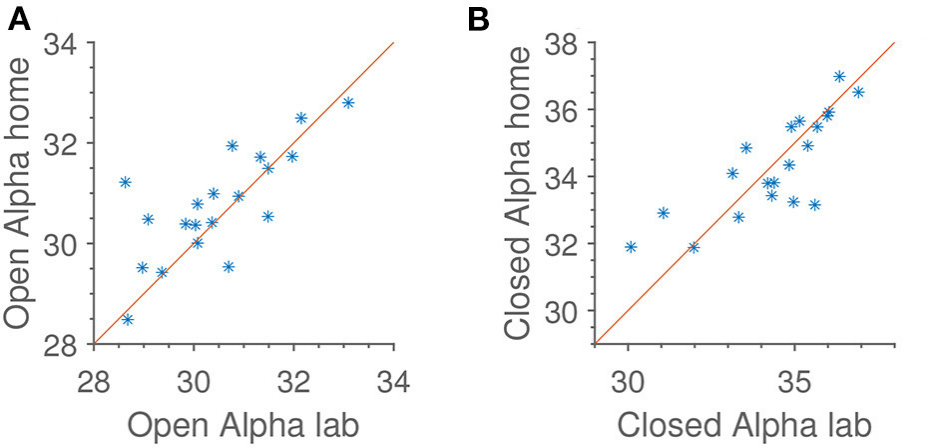
Reproducibility of median alpha power in occipital electrodes, referenced to the average of the rest of the scalp electrodes. Identity line included for reference. Units are dB relative to 1[μ*V*]^2^. **(A)** Open eyes alpha scalp. **(B)** Closed eyes alpha scalp.

In Figure 6 is shown both alpha power for open and closed eyes. As with jaw clenching, we see that alpha powers measured at home and in the lab are very similar, but with some intersubject, reproducible variation.

Not shown in Figure 6 is the subject-wise alpha modulation. When analyzing that, we find an average of 3.6 dB, or slightly more than a two-fold change in power. This is comparable to what is otherwise seen in the literature (Alloway et al., 1997).

### 4.4. EOG Prediction

**Figure 7:**
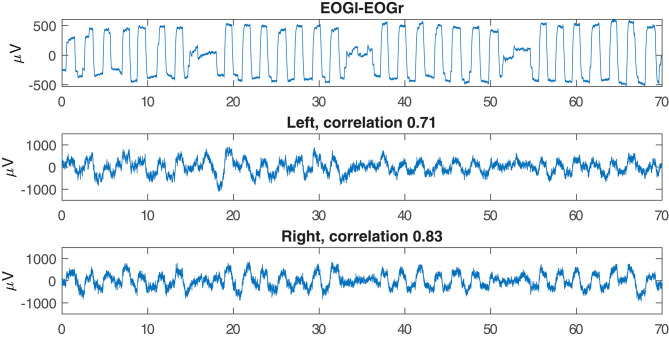
Examples of EOG signals reproduced from the electrodes inside the left or right ear. This example is based on data recorded in the lab.

**Figure 8:**
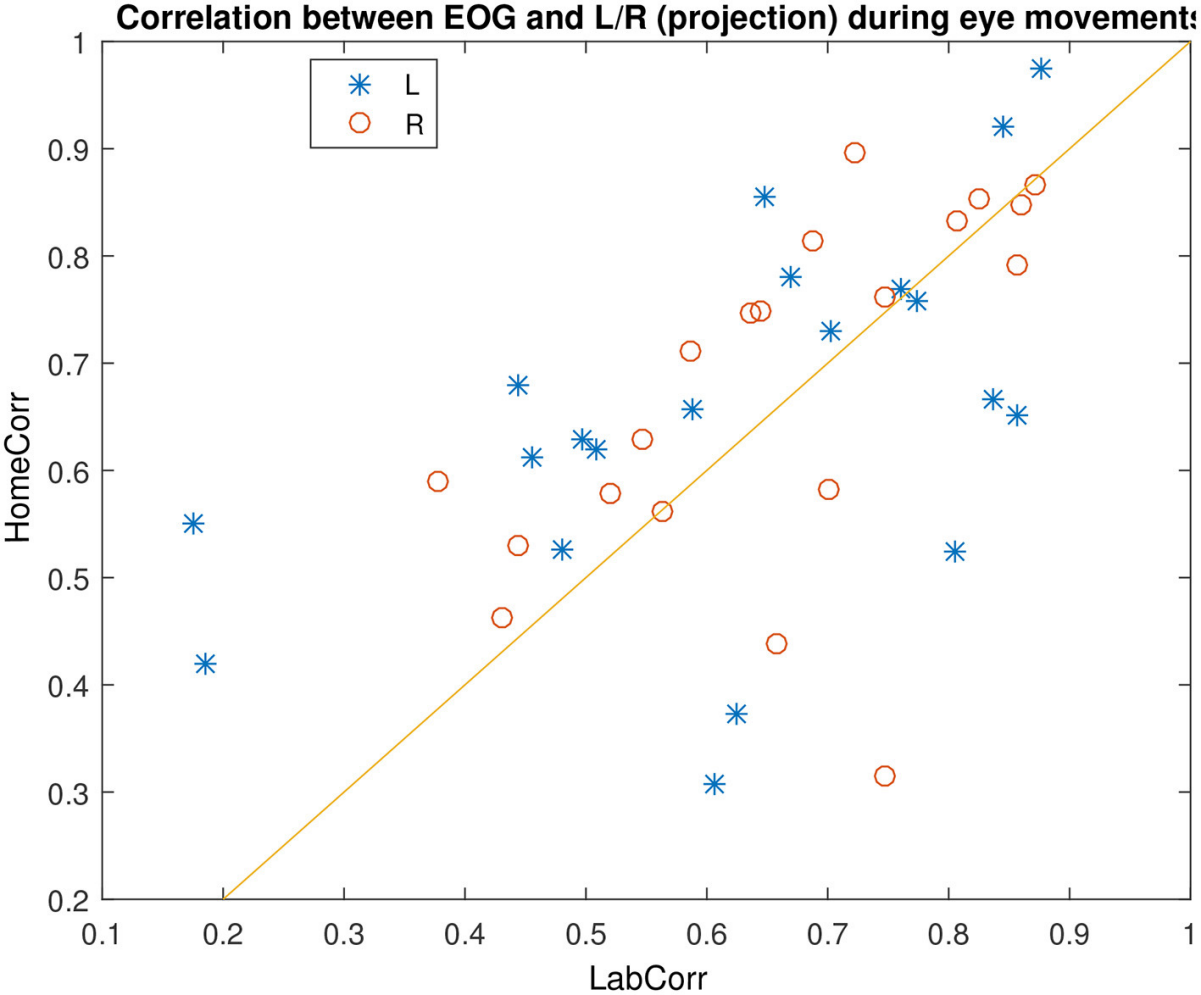
Comparison of EOG prediction quality in the lab and at home, for different subjects. Identity line included for reference.

Figure 7 shows an example of successful EOG prediction in the lab. Figure 8 shows that EOG prediction works to the same extent at home as it does in the lab, though with a great deal of “noise” added.

We find no differences between the two ears. Instead, when calculating the Pearson correlation between prediction quality in left and right ear, we obtain a quite high value of 0.6, meaning that the performance in either ear tends to follow each other. On the other hand, we find very little correlation between performance at home and in the lab on the same day, at 0.11. By investigating the distribution of prediction performances, we find that the major variation in prediction quality is between “high” values that are between 0.5 and 1, and low values which are between 0 and 0.5. It appears that the variation between these two ranges is driven by variations in signal quality in both EOG and ear electrodes. In other words, if the prediction quality is not good (meaning below 0.5), it is most likely due to either many electrodes in the given ear having a bad connection, or one of the EOG electrodes being faulty. This is not particularly correlated between the two locations, which explains the low home vs. lab correlation.

### 4.5. Resting State Power Level

**Figure 9:**
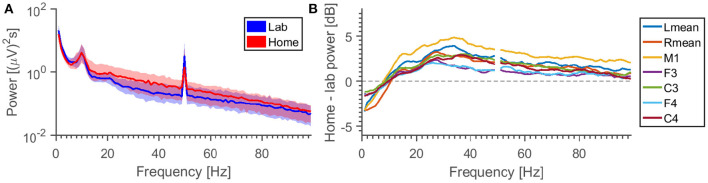
**(A)** Grand average specters, for the M2-C3 EEG channel. For each frequency is calculated the distribution of power densities across all measurements, the region between 25th and 75h percentile is shown (colored area), together with the median (line). **(B)** Difference between median lab and home power specters for different measurement electrodes (all referenced to M2). Note that the specters (excluding the 50 Hz-peak) have been smoothed for added clarity.

Figure 9 shows average power spectra in the EEG for different locations and electrode combinations. There are different observations to be made. (1) The relative difference in power is frequency dependent. For some reason, we see a higher level in the lab setting than home for frequencies below 10 Hz. We hypothesize that this is either due to some special circumstance in our laboratory (since it is unlikely that the noise environments of the test subjects should have some common bias) or long-term settling of the electrodes. (2) The variation in power density is greater than the difference caused by location. (3) The 50 Hz peak behaves differently from the surrounding noise floor—some combinations may have higher 50 Hz power in the laboratory, but lower noise in the surrounding frequency bins.

Performing an ANOVA on the average power between 30 and 100 Hz (excluding 50 Hz), with “subject” and “night” being random factors, the *p*-value for “location” is found to be 5% for all 7 EEG derivations plotted here. If we restrict the data to single EEG channels, the location *p*-value jumps between high (>18%) and low values (<3%).

### 4.6. Variational Analysis

**Figure 10:**
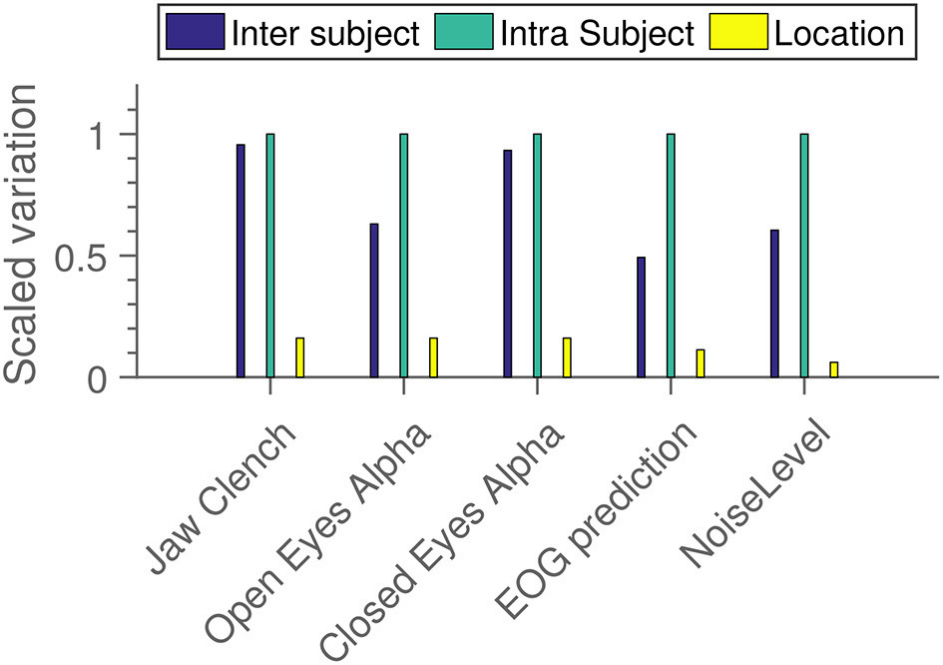
Measures of how much variation is added to the EEG results from different independent variables. Within each subparadigm, the measured standard deviations have been scaled relative to the strongest component.

Figure 10 shows an estimation of the relative contribution to total variation in the data from different sources. We see that in all cases, primary drivers are inter and intra-subject variation, with “location” being mostly a distant third. Note that this is not an estimate of significance—it is absolutely possible for a variable to have a very small, but very probable influence. For instance, it seems quite probable from the results in Figure 9 that location has an influence. But from Figure 10 we see that it contributes less uncertainty to the grand average than both the inter and intra-subject variation.

### 4.7. Significance of Location

In Table 1 is shown differences in mean values and “unexplained error” between laboratory and home recordings. We see that for three of the sub paradigms, the unexplained error is significantly larger at home than in the laboratory, despite the fact that the difference in means is minimal.

**Table 1:** Analysis of location dependence for all sub paradigms.

	**Clench**	**Open-alpha**	**Closed-alpha**	**EOG**	**Resting S.d**.
Location offsets	14.65	0	−0.05	0.05	1.55
Location offsets *p*-values	0.54	0.94	0.15	0.04	0
Unexp. var. diff.	91.03	0.19	0.18	−0.02	1.35
Unexp. var. *p*-values	0	0	0	0.51	0

*The units of offsets and standard deviations are, for column 1: [μV]^2^, and for column 2 and 3: dB relative to 1 [μV]^2^. Column 4 is dimensionless Pearson correlation. Positive values mean that the “home” value is larger*.

It is worth noting that “EOG” is a clear outlier, with a smaller variation in home recordings than in the laboratory, and a quite large *p*-value. This is well explained by the observation made previously—that the main variation in EOG prediction quality is driven by electrode connection, which has no clear pattern between locations.

## 5. Discussion

By analyzing the evoked EEG from a behavioral EEG paradigm performed under many different conditions, we are able to compare the variation in response due to subject difference, location differences and that driven simply by the uncertainty of doing EEG measurements (“intra subject variation”).

We find that for our paradigm, inter subject and intra subject variation contributes more to measurement noise than switching between laboratory and home measurements.

We also find that the signal quality as obtained in the home environment is decent; the rejection rate is actually lower for the home recordings than the lab recordings (7 vs. 11%). The signal to noise ratio is low enough that all parts of the paradigm could be shown to have reproducible results, as presented in Figures 5, 6, 8.

Note that this study does not conclude that location can not have a statistically significant effect on the measured EEG. Instead, we are concluding that the uncertainty added to the results from recording in multiple locations was less than both the intra subject and inter subject variation. Indeed, we do find that in most cases, the amount of unexplained variance (the “fitting error”) is significantly greater (statistically speaking) outside the laboratory than inside it.

We also point out some decisions which had to be made in the design, and which could have changed the results in non-intuitive ways:

1. Some decisions had to be made regarding the definitions of both inter-subject, intra-subject and inter-location variation. Specifically, the choice of modeling “subject” as a *random* factor means that the “shrinkage” caused by “partial pooling” resulted in the inter-subject variability being roughly 85% of what we would have found if “subject” had been modeled as a *fixed* parameter. We have determined this by simply running both analyses.
2. We chose to use the estimated standard deviation of the location offset, rather than the offset itself, to represent the location-based effect. Had we chosen otherwise, the location-based variance would have been estimated at a much lower value.
3. It is likely that “intra-subject variability” could be defined in any number of ways, leading to smaller or larger estimates. However, we do believe that the unexplained variance, which we have chosen here, is a highly relevant quantity for comparison.
4. The “intra-subject” variation is, presumably, quite sensitive to the precise study design. Had the paradigm been longer, resulting in more data for each response calculation, it seems likely that “intra-subject” variation would have been less.

### 5.1. Limitations

In addition to the considerations listed above, there are certain circumstances which limit the generality of our results:

The main things to keep in mind when considering the general relevance of our results are:

1. While the “home” setting is presumably quite varied, the “lab” is not. If the laboratory conditions are somehow exceptional in this study, then that will bias the results. We do not think that this is the case.
2. The way the study was designed, the lab recording always preceded the home-recording. This means that certain time-based effects, such as long-term settling of the electrodes, necessarily influences the two locations differently. It is possible that this is part of the reason for the difference in background power spectra observed in Figure 9.
3. As is common in many neuroscience studies on healthy individuals, our subject cohort was not randomly drawn from the general population. The majority of the participants were engineering students, and it is possible that they would be better than average at carrying out instructions. As to the subset of participants with prior EEG experience, we do not think they biased the results. These subjects were considerably more likely to remove the ear-EEG electrodes between lab and home recordings, which is not the behavior we would expect from participants making an effort to maximize data quality.
4. The study does not include impedance measurements. This was no accident; to the best of our knowledge there are simply no commercially available EEG amplifiers which both have the necessary high input impedance and active shielding required for dry-contact EEG recording as well as the ability to measure electrode impedance. However, as we find that the signal quality (both in terms of automatic data rejection and in terms of recorded responses) is at least as good in the home setting as in the lab, in accordance with the design of the hardware (dry electrode and non-evaporating gels), we are convinced that our electrode connections are stable across both recording sessions.

Finally, we have specifically designed a paradigm which does not rely on precise alignment between recordings and stimuli. While we do believe that such a recording setup could be implemented, we did not attempt to do so in this study, and leave the solution of this problem to future works by either us or our colleagues.

## 6. Conclusion

We present a mixed EEG paradigm which is shown to be insensitive to moving the subject outside the laboratory and out of the direct control of the researcher.

On this basis, we believe that any researchers considering home measurements in a paradigm suited for it (our setup did not require strict control of sensory input, for instance), could do so without worrying that the lack of oversight would unduly contaminate their data. According to our results, if an EEG paradigm is known to work on a broad selection of subjects, it will also work on those subjects in their respective homes.

## Data Availability Statement

The data analyzed in this study were obtained as part of a clinical trial. The following restrictions apply: data can not be shared in any form until 5 years after the end of recordings, due to a combination of Danish regulations on clinical trials and the GDPR. Requests to access these datasets should be directed to Kaare Bjarke Mikkelsen, mikkelsen.kaare@ece.au.dk.

## Ethics Statement

The studies involving human participants were reviewed and approved by Videnskabetiske Komitéer Region Midt. The patients/participants provided their written informed consent to participate in this study.

## Author Contributions

KM designed the study, performed measurements, designed analysis, and wrote the manuscript. PK designed the study, build the hardware, and designed the analysis. YT designed the analysis. CC designed the study and the analysis. All authors contributed to the article and approved the submitted version.

## Conflict of Interest

The authors declare that the research was conducted in the absence of any commercial or financial relationships that could be construed as a potential conflict of interest.
